# Supplementary material for: U–Pb geochronology documents out-of-sequence emplacement of ultramafic layers in the Bushveld Igneous Complex of South Africa
Source: Nat Commun. 2016 Nov 14;7:13385. doi: 10.1038/ncomms13385 (PMC5114581; doi:10.1038/ncomms13385)
Supplement: Supplementary Information — Supplementary Figures 1-3, Supplementary Note 1 and Supplementary References. [file ncomms13385-s1.pdf]

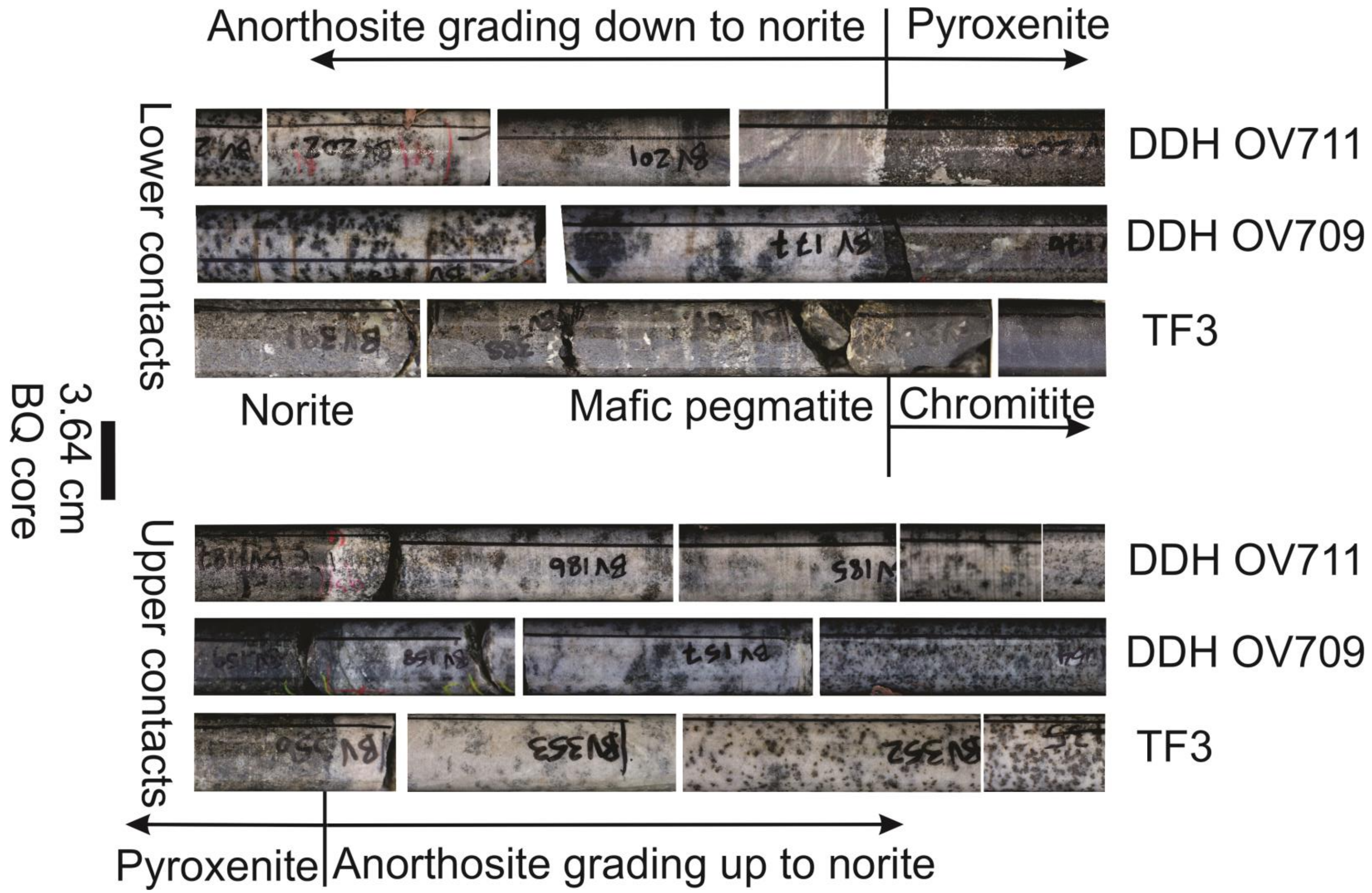
Anorthosite grading down to norite
Pyroxenite
DDH OV711
DDH OV709
TF3
Lower contacts
Norite
Mafic pegmatite
Chromitite
3.64 cm
BQ core
Upper contacts
DDH OV711
DDH OV709
TF3
Pyroxenite
Anorthosite grading up to norite

Supplementary Figure 1. Detailed view of critical textures along the contacts that are shown in Figure 2. These are the same images are are shown in main text Figure 2 but here they have been enlarged and separated from the schematic cross sections. Core samples are 3.64 cm in diameter (BQ size). Note the presence of a chromitite stringer along the upper contacts shown at bottom right, that we interpret as a lag deposit representing the last insoluble residues of otherwise completely assimilated roof-rock norite.

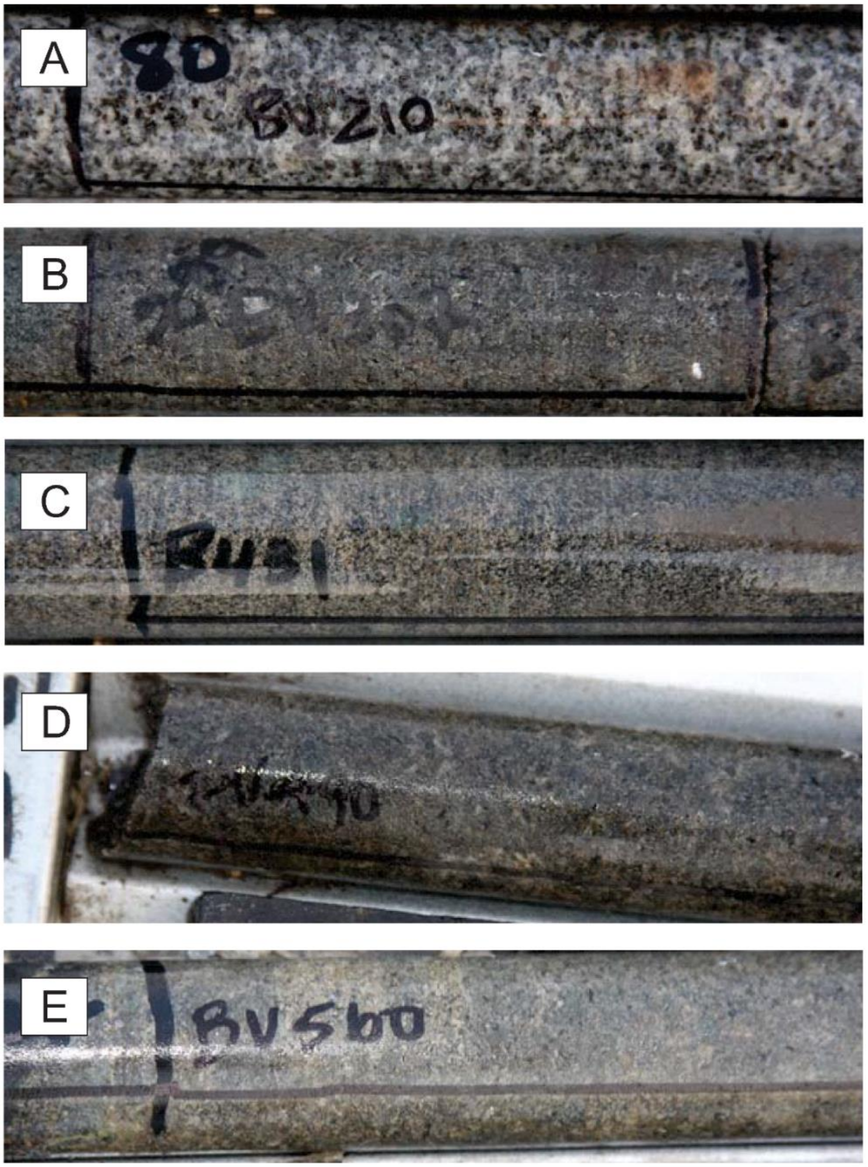

Supplementary Figure 2. Photographs of representative samples of drill core prior to cutting. Cores are 3.64 cm in diameter. A) gabbronorite BV210, representative of composite sample BV204 from between 25.0 and 89.19 m depth in hole TF3. B) feldspathic pyroxenite BV307, representative of composite sample BV305 from between 288.24 and 290.52 m depth in hole TF3. C) feldspathic pyroxenite BV431, representative of composite sample BV431 from between 594.81 and 598.89 m depth in hole TF3. D) feldspathic pyroxenite BV490, representative of composite sample BV486 from between 770.64 and 772.0 m depth in hole TF3. E) feldspathic pyroxenite BV560, representative of composite sample BV561 from between 813.95 and 816.25 m depth in hole TF3.

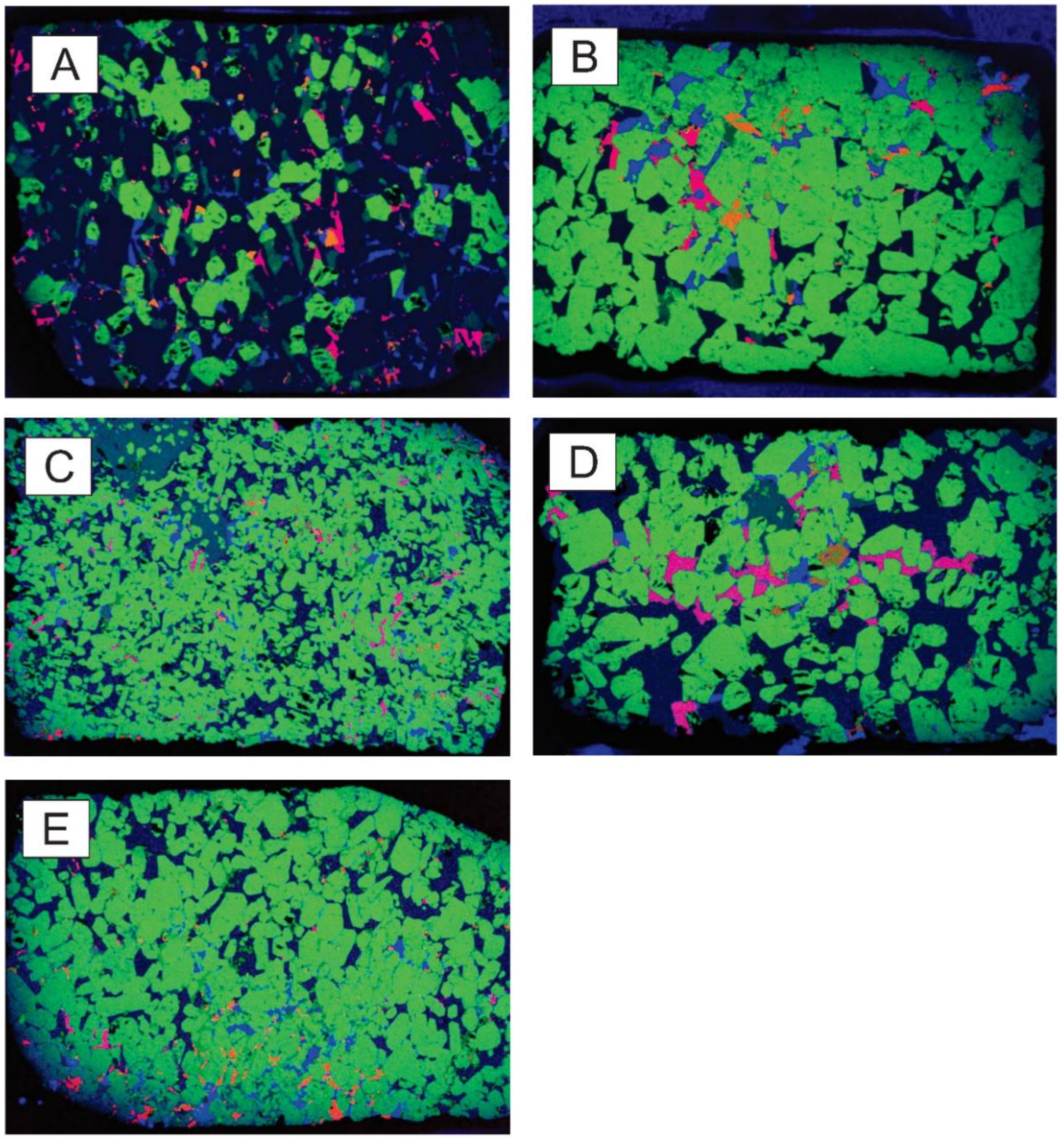

Supplementary Figure 3. False colour RGB X-ray maps of standard polished petrographic thin sections prepared using the Bruker M4 Tornado micro-XRF at CSIRO, Perth, Australia using Mo Kα radiation with a 40 µm spot size. Image processing was done in Corel PHOTO-PAINT X5 by stretching contrasts in individual greyscale element maps and then combining them to the RGB image. Red = K Kα; green = Fe Kα; blue = Si Kα. Mineral colours: dark blue = plagioclase; light blue = quartz; intermediate blue = albite; light green = orthopyroxene; dark green = clinopyroxene; pink = K-feldspar; orange = biotite. Field of view is approximately 4 cm wide. A) gabbronorite BV210 (25.0 m depth in hole TF3), representative of composite sample BV204. B) feldspathic pyroxenite BV307 (289.12 m depth in hole TF3), representative of composite sample BV305. C) feldspathic pyroxenite BV431 (598.0 m depth in hole TF3), representative of composite sample BV431. D) feldspathic pyroxenite BV490 (771.2 m depth in hole

TF3), representative of composite sample BV486. E) feldspathic pyroxenite BV560 (815.0 m depth in hole TF3), representative of composite sample BV561.

Supplementary Note 1.

Sample descriptions. Core photographs of representative materials are shown in Supplementary Figure 1 and X-ray maps of polished thin sections with interpreted mineral distributions are shown in Supplementary Figure 2. Each sample was taken from the vertical diamond drill hole TF3 as a composite of several short pieces of half-core from depth intervals listed below. The hole was collared at 25.692° S, 27.588° E on the western lobe of the RLS (Fig. 1), collecting BQ core 3.64 cm in diameter, to a total depth of 828 m.

The Main Zone is represented by a composite sample comprising half-core segments BV204 (25.00-25.18 m), BV205 (35.00-35.23 m), BV206 (42.00-42.21 m), BV207 (48.81-49.00 m), BV208 (59.81-60.00 m, BV209 (70.00-70.19 m), BV210 (80.00-80.18 m), BV211 (89.00-89.19 m) obtained from indicated depths between 25 and 89.19 m in DDH TF3, called sample BV204 for convenience. The rock is medium-grained equigranular gabbronorite with a weak igneous lamination perpendicular to the core axis defined by parallel alignment of tabular plagioclase crystals.

The Merensky Reef is represented by a composite sample comprising half-core segments BV301 (288.24-288.40 m), BV303 (288.50-288.62 m), BV305 (288.73-288.87 m), BV307 (289.00-289.12 m), BV308 (289.12-289.26 m), BV309 (289.26-289.39 m), BV311 (289.56-289.71 m), BV313 (289.87-290.00 m), BV315 (290.14-290.27 m), and BV317 (290.40-290.52 m) obtained from indicated depths between 288.24 and 290.52 m in DDH TF3, called BV305 for convenience. The rock is medium-grained pyroxenite with an orthocumulate texture defined by orthopyroxene and minor chromite primocrysts up to several mm in size and a matrix of interstitial plagioclase, clinopyroxene, quartz, K-feldspar, biotite, and albite. The UG1 Unit is represented by a composite sample comprising half-core segments BV431 (598.00-598.17 m), BV432 (598.60-598.76 m), and BV433 (598.76-598.89 m), obtained from depths between 594.81 and 598.89 m in DDH TF3, called BV431 for convenience. The rock is fine-grained pyroxenite with an orthocumulate texture defined by < 1 mm orthopyroxene primocrysts and minor cm-sized clinopyroxene oikocrysts in a matrix of interstitial plagioclase, quartz, K-feldspar, biotite, and albite.

The MG4A Unit is represented by a composite sample comprising half-core segments BV486 (770.64-770.82 m), BV488 (770.99-771.14 m), BV491 (771.39-771.61 m), and BV493 (771.79-772.00 m), obtained from indicated depths between 770.64 and 772.0 m in DDH TF3, called BV486 for convenience. The rock is medium-grained pyroxenite with an orthocumulate texture defined by > 1 mm orthopyroxene primocrysts and minor cm-sized clinopyroxene oikocrysts in a matrix of interstitial plagioclase, quartz, K-feldspar, biotite, and albite.

The MG2A Unit is represented by a composite sample comprising half-core segments BV557 (813.95-814.12 m), BV558 (814.20-814.36 m), BV560 (815.00-815.16 m), BV561 (815.66-815.82 m), and BV562 (816.09-816.25 m), obtained from indicated depths between 813.95 and 816.25 m in DDH TF3, called BV561 for convenience. The rock is medium-grained pyroxenite with an orthocumulate texture defined by > 1 mm orthopyroxene primocrysts and minor cm-sized clinopyroxene oikocrysts in a matrix of interstitial plagioclase, quartz, K-feldspar, biotite, and albite.

Supplementary references:

1. Ghiorso, M.S. & Sack, R.O. Chemical mass transfer in magmatic processes IV. a revised and internally consistent thermodynamic model for the interpolation and extrapolation of liquid-solid equlibria in magmatic systems at elevated temperatures and pressures. *Contrib. Mineral. Petrol.* **119**, 197-212 (1995).

2. Asimow, P.D. & Ghiorso, M.S. Algorithmic modifications extending MELTS to calculate subsolidus phase relations. *Amer. Mineral.* **83**, 1127-1132 (1998).

3. Smith, P.M. & Asimow, P.D. Adiabat_1ph: A new public front-end to the MELTS, pMELTS, and pHMELTS models. Geochem. *Geophys. Geosyst*. **6**, art. no. Q02004, doi:10.1029/2004GC000816 (2005).

4. Mattinson, J. M. Zircon U-Pb chemical abrasion ("CA-TIMS") method: combined annealing and multi-step partial dissolution analysis for improved precision and accuracy of zircon ages. *Chem. Geol.* **220**, 47-66 (2005).

5. Jaffey, A.H., Flynn, K.F., Glendenin, L.E., Bentley, W.C. & Essling, A.M. Precision measurements of half-lives and specific activities of 235U and 238U. *Phys. Rev*. C **4**, 1889-1906 (1971).

6. Hiess, J., Condon, D.J., McLean, N. & Noble, S.R. U-238/U-235 systematics in terrestrial uranium-bearing minerals. *Science* **335**, 1610-1614 (2012).

7. Goldberg, L.R., Kercheval, A.N. & Lee, K. t-statistics for weighted means in credit risk modeling. *J. Risk. Finance* 6, 349-365 (2005).
